# Federal Funding and Clinical Trial Sponsorship in Pancreatic Cancer From 2003 to 2022

**DOI:** 10.34172/ijhpm.9255

**Published:** 2026-03-10

**Authors:** Michael A. Mederos, Mark D. Girgis

**Affiliations:** ^1^Department of Surgery, Memorial Sloan Kettering Cancer Center, New York City, NY, USA.; ^2^Department of Surgery, University of California, Los Angeles, Los Angeles, CA, USA.

**Keywords:** Cancer, Funding, Policy, Pancreatic Cancer, Clinical Trials, USA

## Abstract

Pancreatic cancer remains one of the deadliest malignancies, with a persistently low five-year survival rate. The Recalcitrant Cancer Research Act (RCRA) of 2012 is one example of legislation aimed at accelerating research in high-mortality cancers. This study examines long-term trends in federal funding and clinical trial sponsorship for pancreatic cancer over a 20-year period, spanning before and after the RCRA. We conducted a retrospective analysis of National Cancer Institute (NCI) funding data and pancreatic cancer clinical trials registered on ClinicalTrials.gov between 2003 and 2022. Linear regression with pre- and post-2013 comparisons evaluated changes over time. NCI support for pancreatic cancer increased 3.5-fold – from $78 million in 2003 to $250 million in 2022 (inflation-adjusted). The annual growth rate rose from $7.4 million per year before 2013 to $12.2 million per year after 2013. The share of the NCI’s budget allocated to pancreatic cancer also rose from 0.9% to 3.4%, with no significant shift in slope after 2013 (*P*=.98). Federally-funded clinical trials declined sharply before 2013, decreasing by 2.8% per year, and then stabilized after 2013, with a nonsignificant slope of –0.95% per year (interaction *P*=.06). Meanwhile, industry-sponsored trials grew substantially, increasing from 30% to nearly 75%, increasing by 2.0% per year after 2013. Findings were consistent in logistic regression models. These findings suggest that while federal investment in pancreatic cancer research has grown, the expansion of clinical trials has been driven largely by industry, suggesting that federal investment has likely been directed toward foundational and preclinical research. Strengthening public-private collaboration and maintaining federal engagement will be critical to ensuring that research advances align with patient-centered goals.

## Introduction

 Pancreatic cancer is the third leading cause of cancer-related deaths and is projected to surpass colorectal cancer by 2030.^1^ Despite ongoing efforts, the 5-year survival rate has remained dismally low for decades due to late-stage diagnosis, poor understanding of the disease process, and limited treatment options. The discordance in funding and burden of disease for pancreatic cancer and other deadly malignancies is well-recognized.^2,3^ For example, pancreatic cancer was ranked 11th based on funding-to-lethality score, defined as National Cancer Institute (NCI) funding divided by cancer lethality, trailing behind cancers such as breast and melanoma.^2^ To address this critical need, federal legislation such as the Recalcitrant Cancer Research Act (RCRA) of 2012 was introduced, becoming law in January 2013. This bipartisan legislation specifically targeted high-mortality cancers like pancreatic cancer, aiming to direct federal funds toward research and clinical studies for such devastating diseases.

 Having passed a decade since the RCRA’s enactment, this study examines trends in federal funding for pancreatic cancer research and clinical trials over 20 years to evaluate broader trends in the research funding landscape for pancreatic cancer, using the RCRA as a timepoint to evaluate the 10 years before and 10 years after.

## Methods

 Federal funding data were retrospectively sourced from publicly accessible NCI budget archives. The National Institutes of Health (NIH) research support is distributed across multiple institutes, with the NCI serving as the primary source of cancer-specific funding through both investigator-initiated grants and the National Clinical Trials Network. Because the NIH is composed of 27 institutes that fund research through diverse and often nonspecific mechanisms, and because the NCI is the only institute that reports cancer site specific allocations, NCI funding was used as the primary indicator of federal investment in pancreatic cancer research. Pancreatic cancer clinical trials initiated between January 1, 2003, and December 31, 2022, were identified from ClinicalTrials.gov. The clinical trial search strategy is provided in the eMethods (Supplementary file 1). For each trial, phase and funding source were extracted. Funding was categorized as NIH, other U.S. federal agencies, industry (eg, pharmaceutical or device companies), or “other” (eg, universities, private organizations, foundations). Because nearly all federally funded trials were NIH-funded, with only two exceptions, federally funded trials were grouped as NIH-funded. Phase I/II trials were combined with phase II, and phase II/III trials were combined with phase III.

 Year was treated as a continuous variable. Linear regression was used to estimate annual changes in continuous outcomes, including inflation-adjusted NCI funding amounts and the percentage of the NCI budget allocated to pancreatic cancer. All funding figures were inflation-adjusted to January 2024 using the Bureau of Labor Statistics Consumer Price Index. To compare trends before and after the RCRA, the study period was divided at 2013, and interaction models including a Year × Phase term were used to test for differences in slopes. In these models, the coefficient for year represented the annual rate of change during 2003–2012, and the sum of the year and interaction coefficients represented the annual rate of change during 2013–2022.

 The annual number of clinical trials outcome was analyzed using quasi-Poisson regression with a log link to account for overdispersion. These models also included a Year × Phase interaction to evaluate whether the rate of change differed after 2013. Proportional outcomes, including the proportion of trials funded by NIH, industry, or other sponsors, were analyzed using linear regression for interpretability and consistency with graphical presentation. Binomial logistic regression was performed as a sensitivity analysis. Statistical significance was defined as a 2-sided *P* <.05. All analyses were conducted in R (version 2024.04.2+764).

## Results

**Figure 1:**
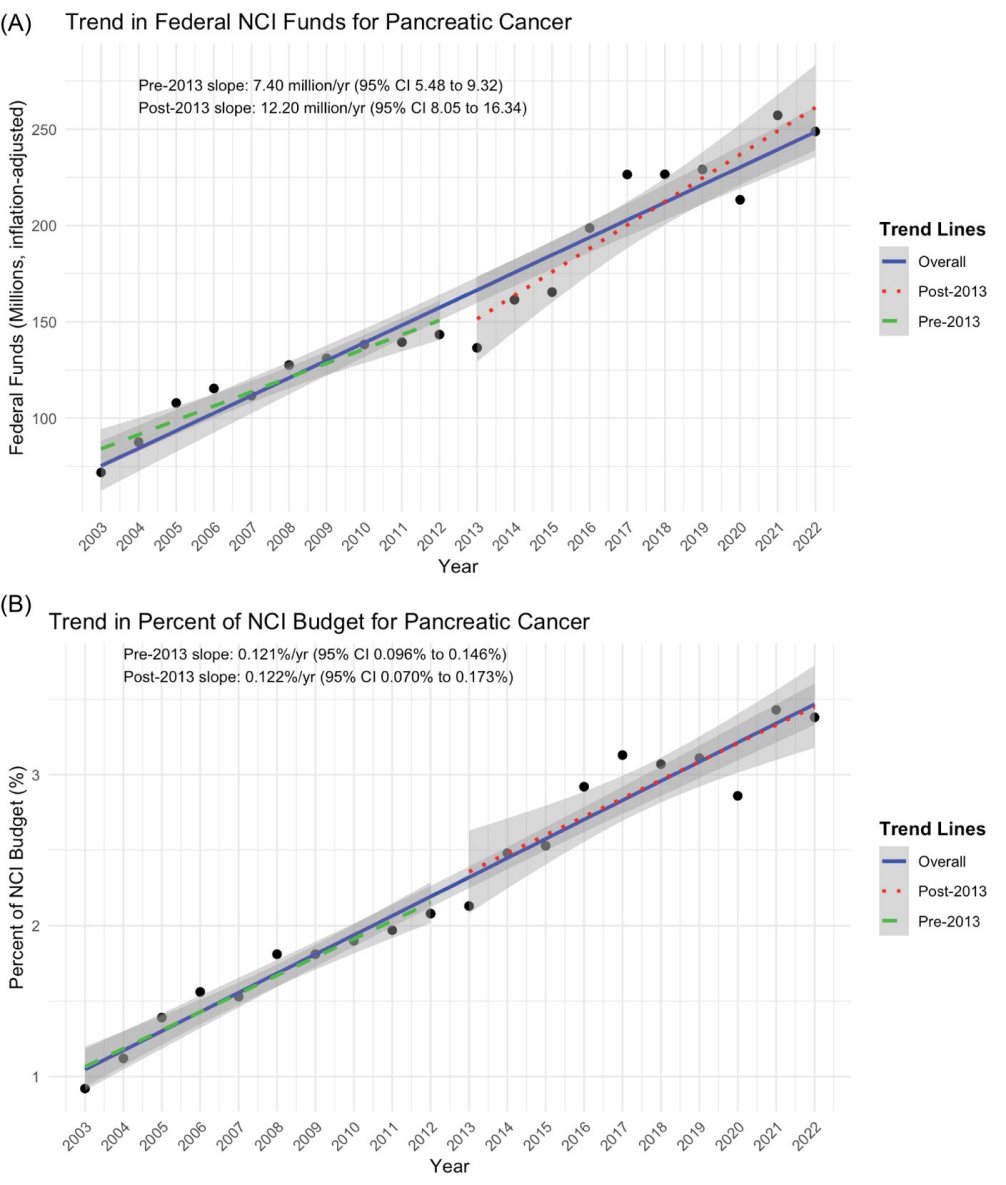
(A) Trend in National Cancer Institute funding (2003-2022). (B) Trend in Percent Budget Allocation for Pancreatic Cancer (2003-2022). Solid blue line represents the trend for the entire study period. The green dashed line and red dotted line represent the trend lines for 2003 to 2012 and 2013 to 2022, respectively. The gray shaded areas represent 95% confidence intervals. Abbreviations: NCI, National Cancer Institute; CI, confidence interval.

 Over the 20-year study period, total inflation-adjusted NCI funding for pancreatic cancer research rose 3.5-fold, from $78 million in 2003 to approximately $250 million in 2022 (Figure 1A). The rate of funding growth varied between the two halves of the study period. Annual increases were $7.4 million between 2003–2012 and $12.2 million between 2013–2022 (*P* =.03). This represented a 3.7-fold increase in the proportion of the NCI budget dedicated to pancreatic cancer, from 0.9% to 3.4% (Figure 1B). The NCI budget share allocated to pancreatic cancer increased by 0.12 percentage points per year in both periods (*P* =.98), and there was no significant level change at 2013 (*P* =.78).

**Figure 2:**
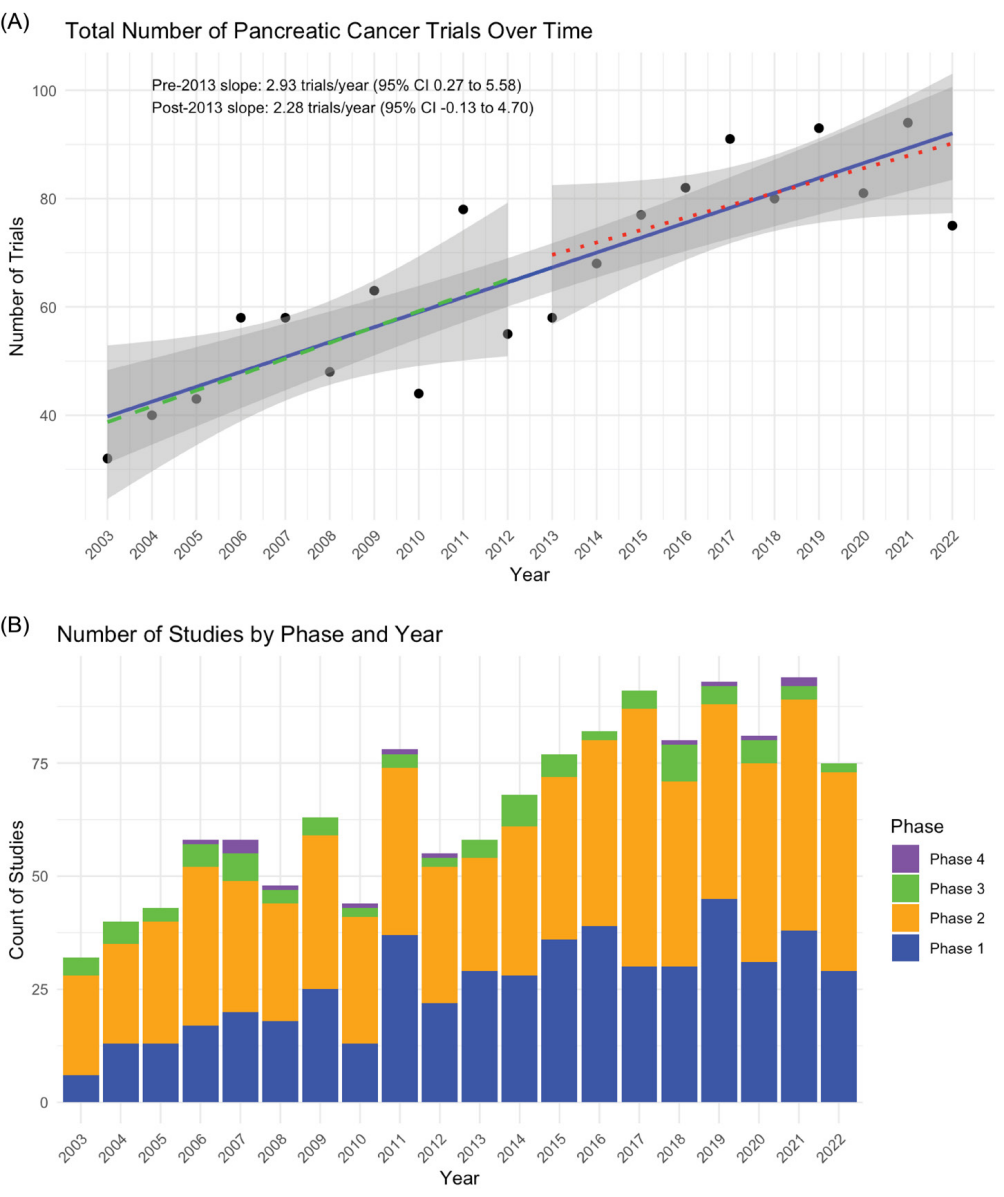
(A) Trend in Clinical Trials (2003-2022). Solid blue line represents the trend for the entire study period. The green dashed line and red dotted line represent the trend lines for 2003 to 2012 and 2013 to 2022, respectively. The gray shaded areas represent 95% confidence intervals. (B) Clinical Trials by Phase (2003-2022). Annual count of trials by phase (I, II, III, IV). Bars represent total trials per year; shading indicates the distribution of trial phases. Abbreviation: CI, confidence interval.

 From 2003 to 2022, 1318 pancreatic cancer clinical trials were initiated, ranging from 32 trials in 2003 to a peak of 94 trials in 2021 (Figure 2A). In quasi-Poisson models, the number of trials increased by 5.8% per year during 2003-2012 (incidence rate ratio [IRR], 1.06; *P* =.01). There was no significant level change at 2013 (IRR, 1.33; *P* =.30), and the slope after 2013 did not differ significantly from the pre-2013 period (interaction IRR, 0.97; *P* =.28). These results indicate a steady rise in trial volume over time without evidence of an inflection at 2013. The overall increase in trials was driven primarily by a surge in early-phase trials (phases I and II) (Figure 2B). The number of trials that received at least partial funding from the federal government remained stable throughout the study period (slope = 0.03, *P* = 0.87) (Figure S1A). Most federally funded studies also received supplementary funding from industry or other sources; trials solely funded by federal sources were relatively few (Figure S1B).

**Figure 3:**
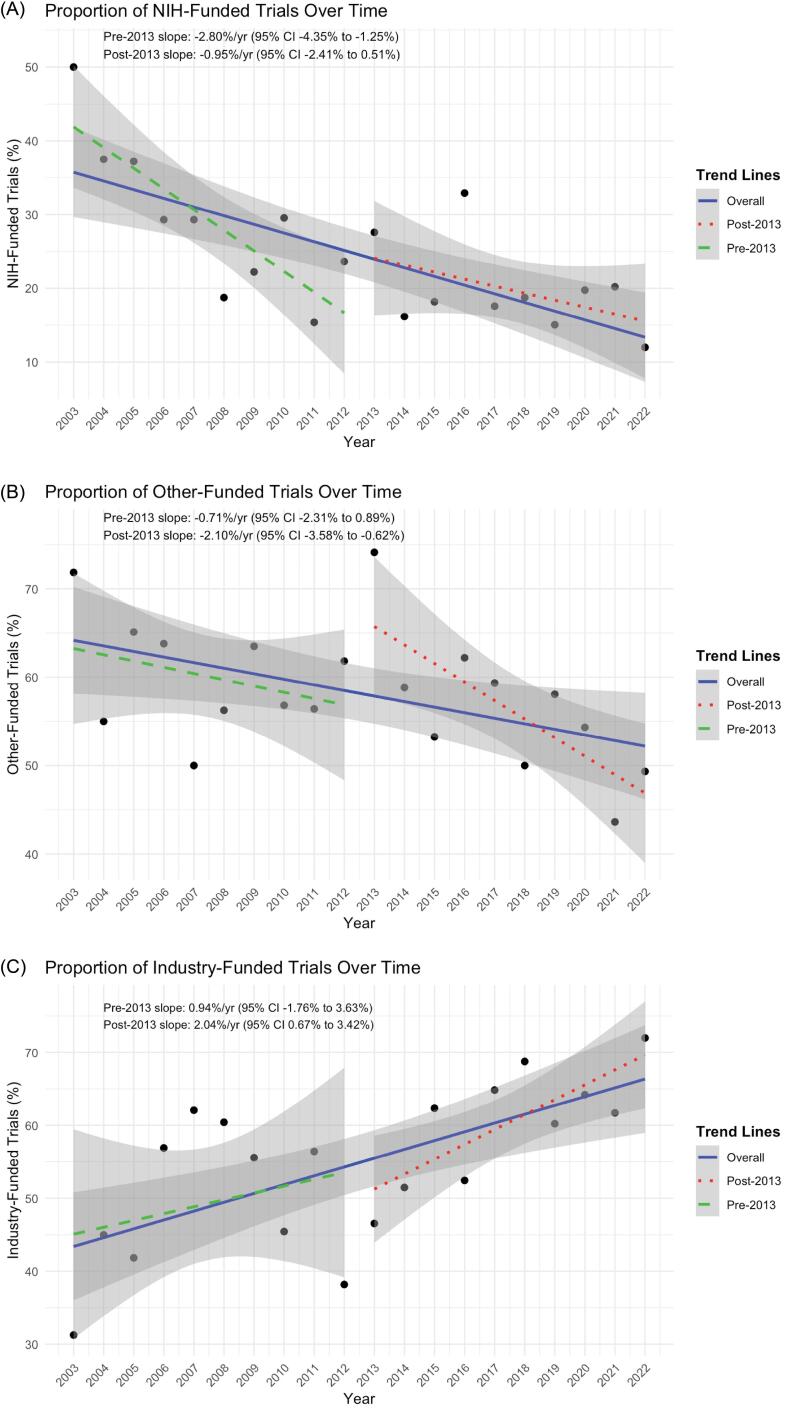
(A-C) Proportional Change in Funding Sources for Clinical Trials (2003-2022). Solid blue line represents the trend for the entire study period. The green dashed line and red dotted line represent the trend lines for 2003 to 2012 and 2013 to 2022, respectively. The gray shaded areas represent 95% confidence intervals. Abbreviations: NIH, National Institutes of Health; CI, confidence interval.

 Federally funded trials remained relatively stable in number but declined as a proportion of all trials (Figure 3A). The proportion of federally sponsored trials declined significantly from 2003-2012 (β = –0.028 per year, *P* <.001). There was no significant level shift at 2013 (*P* =.43). Although the post-2013 slope was numerically less negative (interaction β = +0.0185, *P* =.062), the slope difference did not reach statistical significance, indicating that the decline in NIH-funded trials did not meaningfully change after 2013.

 The proportion of “other”-funded trials declined over time (slope = -0.63) (Figure 3B). During 2003–2012, the proportion decreased by 0.7 percentage points per year (β = –0.007), and by 2.1 percentage points per year after 2013, though the slope difference was not significant (interaction *P* =.16). A higher baseline proportion was observed in 2013 (β = 0.235; *P* =.04).

 Industry sponsorship showed a gradual upward trend. During 2003–2012, the proportion of industry-funded trials increased slightly but not significantly (β = 0.009 per year, *P* =.33), and there was no level change at 2013 (*P* =.34). Although the slope during 2013–2022 was numerically higher (0.020 per year), the slope difference was not significant (interaction β = 0.011, *P* =.41).

## Discussion

 This study showed an overall upward trend in federal funding for pancreatic cancer, with a corresponding increase in the proportion of NCI’s budget allocated to the disease. Post-2013, there was an acceleration in both federal funding and industry participation, possibly related to policy changes such as the RCRA and initiatives like the Cancer Moonshot. The shift toward industry funding may also reflect a growing emphasis on personalized medicine and targeted therapies, which attract industry collaboration.

 There was a steady increase in clinical trials related to pancreatic cancer throughout the study period. Interestingly, the rise in federal funding did not correspond to an increase in federally funded trials. Instead, the surge in trials was primarily driven by industry funding. Industry-funded trials increased from 30% to nearly 75%, consistent with trends seen in other studies.^4,5^ It is likely that appropriated federal funds for pancreatic cancer were likely allocated to pre-clinical and basic science research. This important work may serve as the foundation for the industry-funded clinical trials. Therefore, the increased discretionary funding may be partially responsible for the observed increase in clinical trials regardless of funding source. This general trend may also explain a 40% reduction in NIH-funded trials from 2005 to 2015 that has been previously noted.^6^ Additionally, industry payments to NCI-designated cancer centers reached $3 billion in 2019.^7^ The partnership between industry, government, as well as academia, is crucial for advancing cancer research, especially for challenging diseases like pancreatic cancer.

 However, the growing reliance on industry funding raises concerns about potential biases. Industry-funded trials may prioritize profit-driven motives over patient outcomes, necessitating ongoing federal oversight to ensure the public interest remains central in cancer research. Despite this, the study underscores the value of partnerships between public and private sectors. More research is needed to understand the impact of these partnerships on the efficiency, cost, and outcomes of pancreatic cancer research.

 This study has several limitations. Federal funding estimates relied on NCI budget reports, which are the only NIH source that provides cancer site specific allocations. As a result, other NIH mechanisms that may indirectly support pancreatic cancer research were not captured. Clinical trial sponsorship data from ClinicalTrials.gov may include reporting inconsistencies. Finally, because this was an ecological analysis using aggregate data, causal conclusions regarding the impact of any specific policy cannot be drawn.

## Conclusion

 In the two decades examined, federal funding for pancreatic cancer increased significantly, alongside a rise in industry-funded trials. While the RCRA and subsequent initiatives have likely contributed to this growth, the shifting landscape toward industry-led trials underscores the need for balanced oversight. Continued collaboration between federal agencies, industry, and academia will be essential in driving research that can meaningfully improve outcomes for pancreatic cancer patients.

## Disclosure of artificial intelligence (AI) use

 AI tools (eg, ChatGPT) were used during the preparation of this manuscript solely for editing purposes, including grammar, syntax, and structural clarity. All content, data interpretation, and scientific conclusions were generated and reviewed by the authors.

## Ethical issues

 Ethics approval was not required for this study, as all data were obtained from publicly available sources.

## Conflicts of interest

 Authors declare that they have no conflicts of interest.

## Supplementary files


Supplementary file 1 contains Figure S1A-B and eMethods.
