# Supplementary material for: Federal Funding and Clinical Trial Sponsorship in Pancreatic Cancer From 2003 to 2022
Source: Int J Health Policy Manag. 2026 Mar 10;15:9255. doi: 10.34172/ijhpm.9255 (PMC13145243; doi:10.34172/ijhpm.9255)
Supplement: Supplementary file 1 — contains Figure S1A-B and eMethods. [file ijhpm-15-9255-s001.pdf]

Article title: Federal Funding and Clinical Trial Sponsorship in Pancreatic Cancer From 2003 to 2022

Journal name: International Journal of Health Policy and Management (IJHPM)

Authors' information: Michael A. Mederos[1], Mark D. Girgis[2]*

[1]Department of Surgery, Memorial Sloan Kettering Cancer Center, New York City, NY, USA.

[2]Department of Surgery, University of California, Los Angeles, Los Angeles, CA, USA.

***Correspondence to:** Michael A. Mederos; Email: mmederos.md@gmail.com

Citation: Mederos MA, Girgis MD. Federal funding and clinical trial sponsorship in pancreatic cancer from 2003 to 2022. Int J Health Policy Manag. 2025;14:9255. doi:10.34172/ijhpm.9255

Supplementary file 1

eMethods:

Clinicaltrials.gov search criteria:

Condition/disease: “Pancreatic Cancer”

Location: “United States”

Study status: “all studies”

Age: “all ages”

Accepts healthy volunteers: left blank

Study Phase: Early phase 1, phase 1, phase 2, phase 3, phase 4

Study type: N/A

Study results: N/A

Study documents: N/A

Funder type: include all by default.

Date range:
Study start: 1/1/2003 to 12/31/2022
Primary completion: N/A
First posted: N/A
Results first posted: N/A
Last update posted: N/A
Study completion: N/A

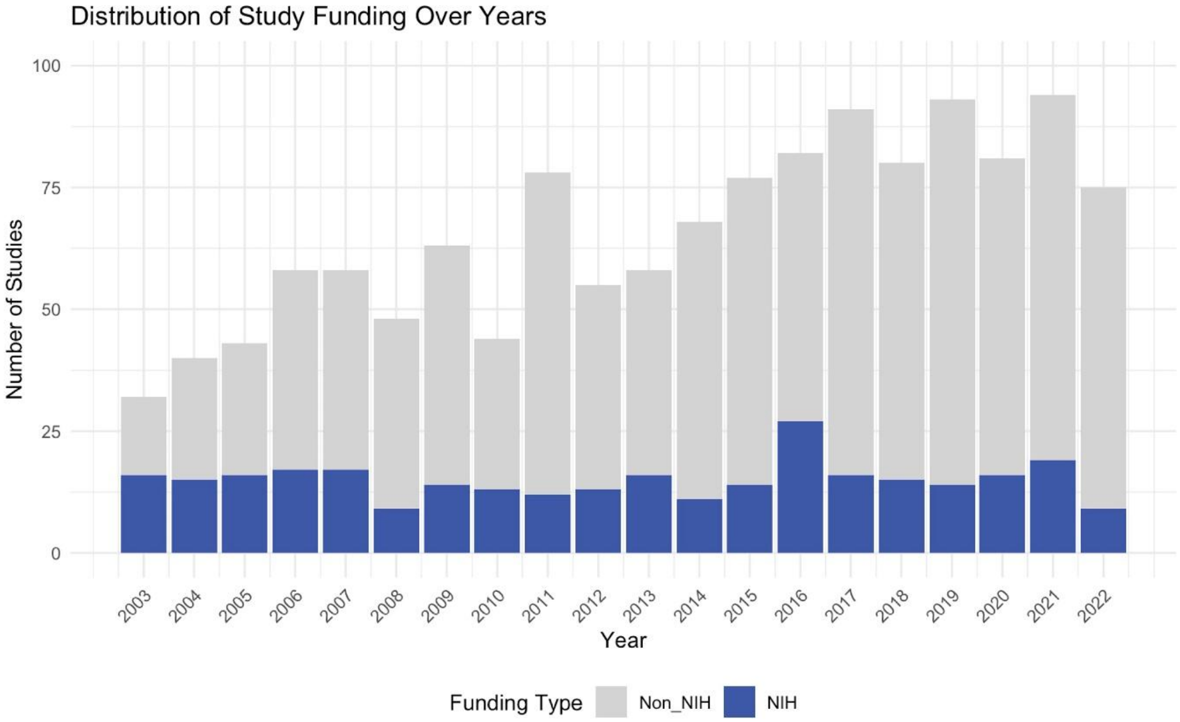

Figure S1A. Studies receiving National Institutes of Health funding (2003-2022)

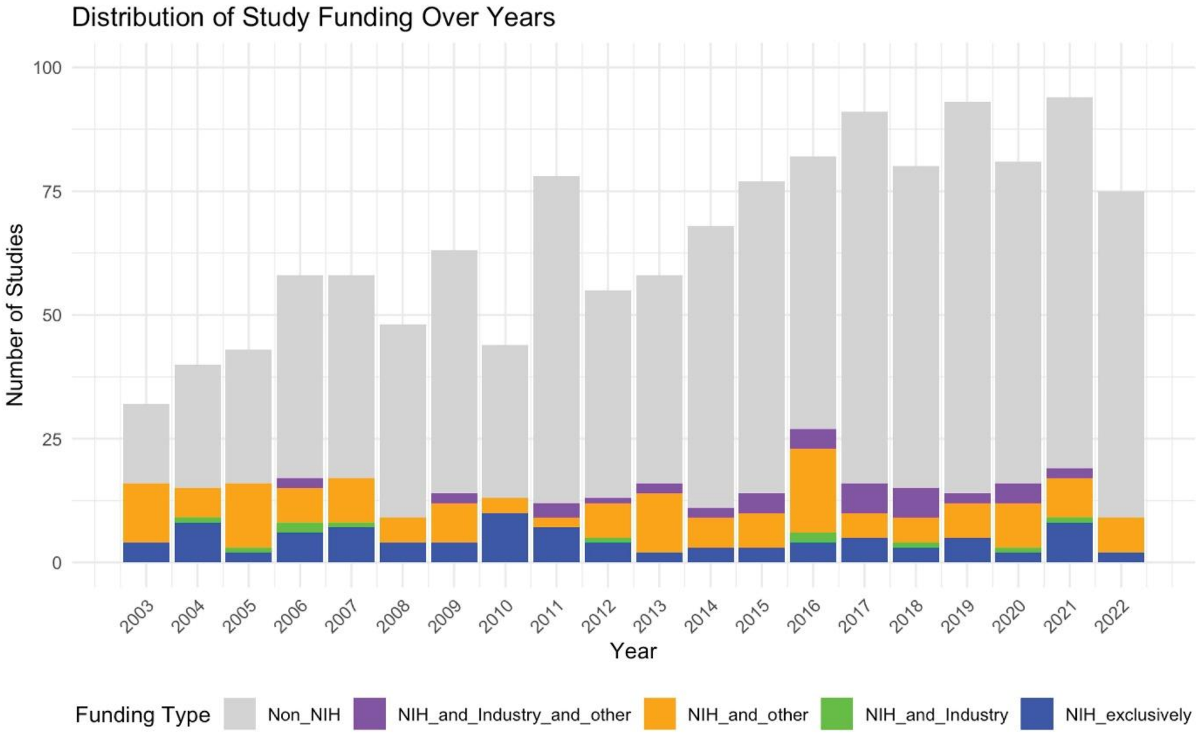

Figure S1B. Collaboration of funding sources in federally-funded studies
